# Supplementary material for: The Pseudomonas putida type VI secretion systems shape the tomato rhizosphere microbiota
Source: ISME Commun. 2025 Sep 9;5(1):ycaf158. doi: 10.1093/ismeco/ycaf158 (PMC12482989; doi:10.1093/ismeco/ycaf158)
Supplement: Supplementary_Data_T6SS_Rhizobiota_ycaf158 [file supplementary_data_t6ss_rhizobiota_ycaf158.pdf]

SUPPLEMENTARY INFORMATION FOR

The *Pseudomonas putida* Type VI Secretion Systems Shape the Tomato Rhizosphere Microbiota

David Vázquez-Arias[1], Cristina Civantos[2], David Durán-Wendt[1], Adrián Ruiz[2], Rafael Rivilla[1], Marta Martín[1]* and Patricia Bernal[2]*

[1]Departamento de Biología, Facultad de Ciencias, Universidad Autónoma de Madrid, Darwin, 2, 28049 Madrid, Spain.

[2]Departamento de Microbiología, Facultad de Biología, Universidad de Sevilla, Avenida de la Reina Mercedes, 6, 41012 Sevilla, Spain.

*For correspondence email: m.martin@uam.es and pbernal@us.es

This file includes:

Figure S1 to S5

Tables S1 to S6

Supplementary references

SUPPLEMENTARY INFORMATION FIGURES

Figure S1

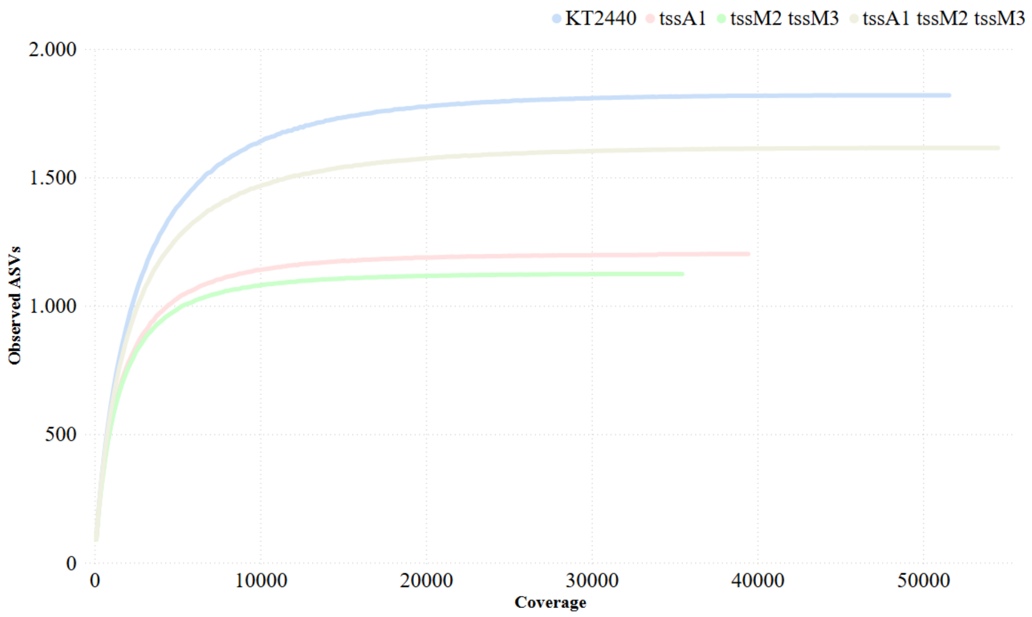

Supplementary Figure 1 | Bacterial 16S rRNA Gene Amplicon Sequence Variant (ASV) Rarefaction Curves. The plot represents the relationship between the number of sequenced reads (x-axis) and the corresponding observed diversity of bacterial ASVs (y-axis) for each of the 4 biological samples analysed. Each individual curve represents the combined sequence data obtained from the technical replicates of a single biological sample. All four curves plateaued, suggesting that the sequencing depth of our analysis was sufficient.

Figure S2

A)

VgrG2-VgrG3-VgrG4-VgrG5 alignment

Percent of Identity of VgrG2 with	**VgrG3**	VgrG4	VgrG5
	**96.31%**	73.68%	70.59%

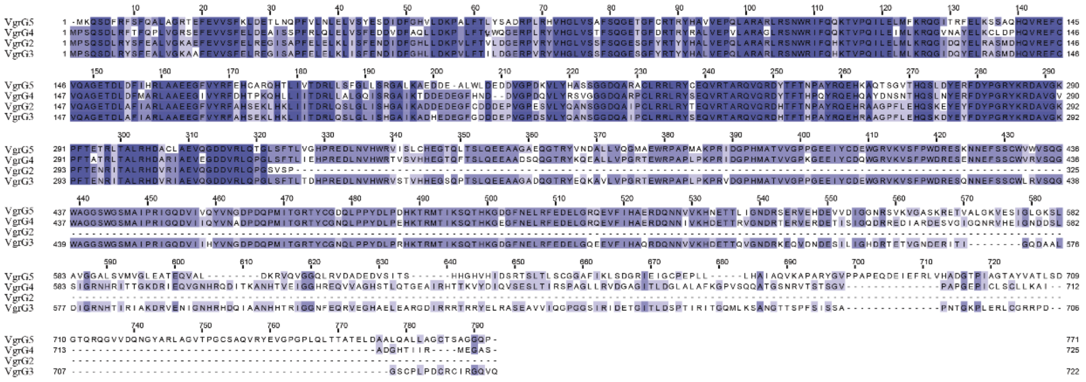

B)

TssC3-TssC2 alignment

Percent of Identity of TssC2 with TssC3 - **90.89%**

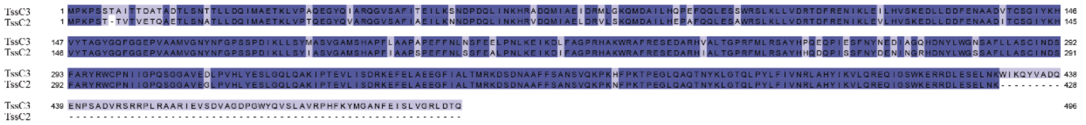

Supplementary Figure 2 | Multiple Sequence Alignment (MSA) of VgrG and TssC proteins of *P. putida* KT2440 performed with Clustal Omega (Maderia et al., 2022) and visualised by Jalview, coloured by percentage of identity. Simplified percent identity matrices (generated by Clustal 2.1) are included for each alignment (boxed in grey). **A)** VgrG proteins have a conserved N-terminal region (~1-550 aa), which allows interaction with other structural components of the system, primarily with the Hcp ring (Allsopp and Bernal 2023). The aligned VgrG proteins (VgrG2, VgrG3, VgrG4 and VgrG5) all contain the highly conserved region, but VgrG2 presents a premature stop codon that stops translation after amino acid 325. The identity between VgrG3 and the truncated VgrG2 is extremely high (>96%). **B)** Similarly, the identity between TssC3 and TssC2 is high (>90%) but TssC2 does not have the last 64 amino acids from a total of 495.

Figure S3

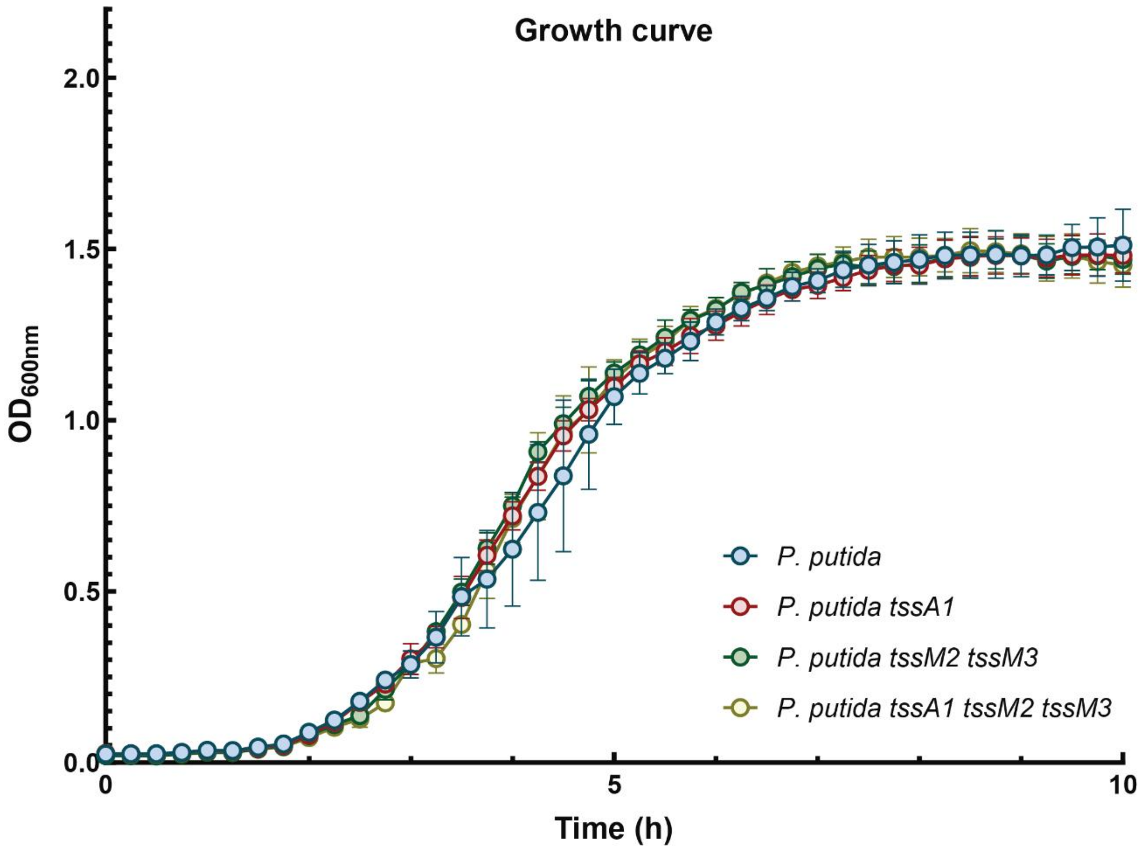

Supplementary Figure 3 | Growth curve of *P. putida* wildtype and mutant strains. Overnight cultures grown on LB Rif were adjusted to OD_{600} of 0.05 in fresh LB media. OD_{600} was measured for a total of 10 hours.

Figure S4

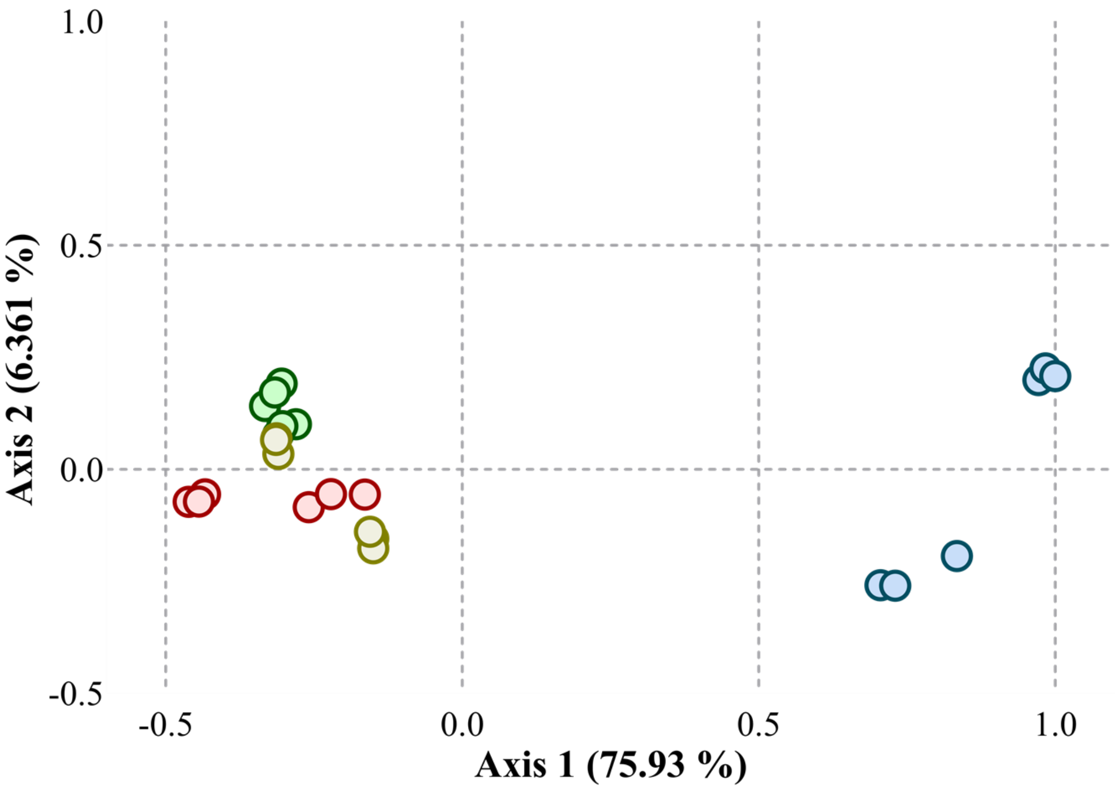

Figure S4. Analysis of rhizosphere microbiome diversity without KT2440 ASV. Principal Coordinates analysis (PCoA) of three 16S rRNA gene replicates from each soil sample using Bray-Curtis distances. Axis 1 explains 76 % of the observed variance.

Figure S5

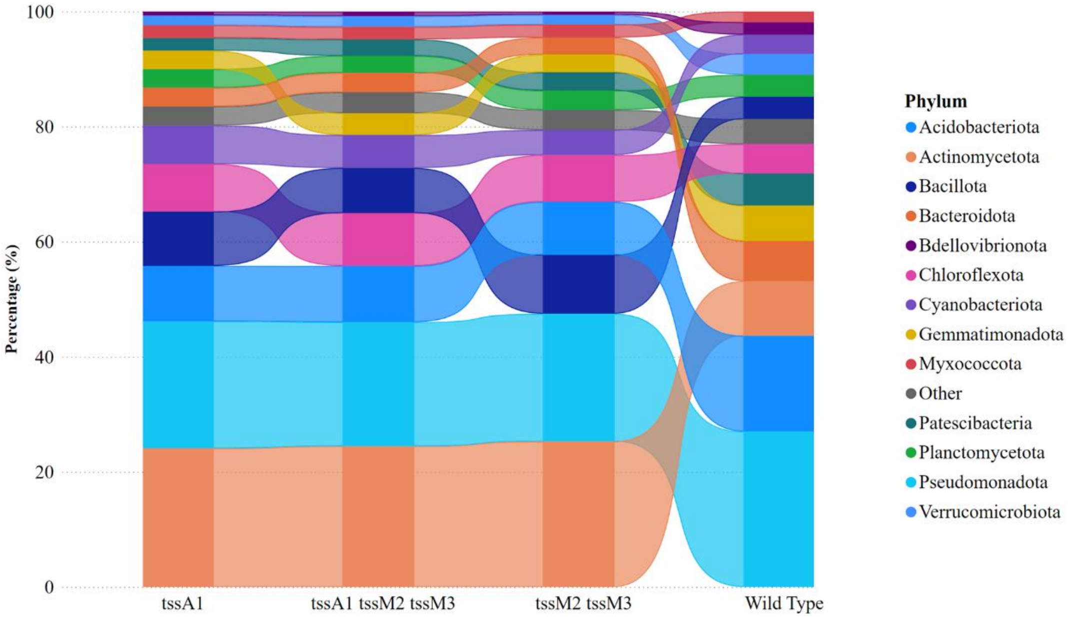

Supplementary Figure 5 | Relative abundance of Bacterial Phyla in Inoculated Soil Sample. The barplot represents the average relative abundance of bacterial ASVs aggregated at the phylum level, as observed in each inoculated soil sample two weeks post-inoculation. Each bar represents a specific soil sample inoculated with the wildtype, the *tssA1*, the *tssM2 tssM3* or the *tssA1 tssM2 tssM3* mutant strains. The height of the coloured segments within each bar indicates the average proportion of ASVs assigned to different bacterial phyla within that sample. To facilitate the comparison of individual taxa across the different samples, ribbons connect the segments representing the same phylum in adjacent bars.

SUPPLEMENTARY INFORMATION TABLES

Table S1. Bacterial strains used in this study. The antibiotic resistance markers are identified as follows: Amp, ampicillin; Km, kanamycin; Gm, gentamicin, Sm, streptomycin; Nal, nalidixic acid; Pip, piperacillin and Rif, rifampicin.

Name	Description	Source
Escherichia coli		
DH5α	F^- *end*A1 *gln*V44 *thi*-1 *rec*A1 *rel*A1 *gyr*A96 *deo*R *nup*G *pur*B20 φ80d*lacZ*ΔM15 Δ(*lac*ZYA-*arg*F*)*U169 *hsd*R17($r_K^-m_K^+$) λ^-, Nal^R	[1]
CC118λpir	*ara*D Δ(*ara*, *leu*) Δ*lac*Z74 *pho*A20 *gal*K *thi*-1 *rsp*E *rpo*B *arg*E *rec*A1 λ*pir*, Rif^R	[2]
HB101	*supE44 hsdS20 recA13 ara-14 proA2 lacY1 galK2 rpsL20 xyl-5 mtl-1*, Sm^R	[3]
Pseudomonas putida		
KT2440R	Wild type strain, Rif^R	[4]
KT2440R *tssA1*	Markerless mutant in the *tssA1* (PP3088) gene, disabling the K1-T6SS; Rif^R	[5]
KT2440R *tssM2 tssM3*	Markerless KT2440R null mutant in the *tssM2* (PP4071) and *tssM3* (PP2627) gene, disabling the K2-and K3-T6SSs, respectively; Rif^R	This study
KT2440R *tssA1 tssM2 tssM3*	Markerless KT2440R null mutant in the *tssA1*, *tssM2* and *tssM3* genes	[5]
Plant pathogens		
Xanthomonas campestris pv. campestris IVIA 2734-1		[5]

Table S2. Plasmids used in this study. The antibiotic resistance markers are identified as follows: Amp, ampicillin; Km, kanamycin; Gm, gentamicin, Sm, streptomycin, Cm, chloramphenicol, Pip, piperacillin and Rif, rifampicin.

Name	Description	Source
pCR-BluntII-TOPO	Cloning vector, ColE1 *ori*, Km^R	Invitrogen
pRK600	Helper plasmid, ColE1 *ori, mob*RK2, *tra*RK2, Cm^R	[6]
pKNG101	Gene replacement suicide vector, R6K *ori*, *sacB;* Sm^R	[7]
pKNG101-*tssM2*	1.6-Kb PCR fragment containing the regions upstream and downstream *tssM2* cloned in pKNG101 (*Xba*I-*Bam*HI) for double recombination; when inserted into the chromosome and the plasmid cured, the strain is a *tssM2* mutant; Sm^R	[5]
pKNG101-*tssM3*	1.6-Kb PCR fragment containing the regions upstream and downstream *tssM3* cloned in pKNG101 (*Xba*I-*Bam*HI) for double recombination; when inserted into the chromosome and the plasmid cured, the strain is a *tssM3* mutant; Sm^R	[5]
pRL662-*gfp2*	pRL662 [8] derivative expressing GFP2; Gm^R	Erh-Min Lai collection

Table S3. Oligonucleotide primers used in this study. The "Brief description" column provides basic information on the primer design (restriction enzyme used for cloning, encoded protein, forward or reverse orientation of the primer (F or R). Primers marked with UP or DOWN were used to construct mutant strains. Seq stands for sequencing primers. Primers tagged with 16S were used to amplify the V3-V4 regions of the 16S rRNA gene (**N**: any nucleotide; **W**: A or T; **H**: A or T or C; **V**: G or A or C). Sequences recognised by restriction enzymes are highlighted in lower case.

Number	Brief description	Sequence (5´-3´)
P1	XbaI.*tssM2*.UP.F	GAGAGtctagaGACGCGCCGAGCCATCTT
P2	*tssM2*.UP.R	CTACATGAC<u>TTGATTCATCGAGGCTCC</u>
P3	*tssM2*.DOWN.F	ATGAATCAA<u>GTCATGTAGGCAGGAGGC</u>
P4	BamHI.*tssM2*.DOWN.R	TTCAAggatccGCGTGAACGCTCGTTACA
P5	*tssM2*.UP.F.Seq	TTGAGCTGGAGCGCCTGTTG
P6	*tssM2*.UP.R.Seq	CGCGAGATCCGCTGGATAAC
P7	XbaI.*tssM3*.UP.F	AGGAAtctagaACGACGCTACCGGCTACC
P8	*tssM3*.UP.R	TCATAGTCGTTGATTCATCGAGGCTCC
P9	*tssM3*.DOWN.F	ATGAATCAACGACTATGAACCTCGTCA
P10	BamHI.*tssM3*.DOWN.R	ACCTTggatccTGAGCTGACGCTGCACAT
P11	*tssM3*.UP.F.Seq	GAGCGCCTGCTAGGCAAGTAC
P12	*tssM3*.UP.F.Seq	TGCGCGTCAACGGTATGTCG
P13	TOPO.F.Seq	GTAAAACGACGGCCAG
P14	TOPO.F.Seq	CAGGAAACAGCTATGAC
P15	KNG.F.Seq	CATATCACAACGTGCGTGGA
P16	KNG.R.Seq	CCCTGGATTTCACTGATGAG
P17	341F-16S	CCTACGGGNGGCWGCAG
P18	805R-16S	GACTACHVGGGTATCTAATCC

Table S4. ASV count table. The table is provided as a separate Excel file due to its complexity. First column (A): ASV code; second column (B): taxonomy; third column (C): mean of abundance of the different ASV in the plant inoculated with the wildtype strain; fourth column (D): mean of abundance of the different ASV in the plant inoculated with the single *tssA1* mutant; (E): mean of abundance of the different ASV in the plant inoculated with the double *tssM2 tssM3* mutant; (F): mean of abundance of the different ASV in the plant inoculated with the triple *tssA1 tssM2 tssM3* mutant.

Table S5. Analysis of the influence of the *P. putida* T6SSs in the bacterial composition of tomato rhizosphere. Assessment of the impact of T6SS mutations on the overall structure of the tomato rhizosphere microbiome performed by PERMANOVA (Permutational Multivariate Analysis of Variance) Adonis using distance matrices. The analysis partitions the total variation in the microbial community data to determine the proportion explained by the factor 'Strain' (wildtype and three T6SS mutants) versus unexplained residual variation. The results demonstrate a highly significant effect of strain (pseudo-F = 23.06, $p = 0.001$), accounting for a substantial 77.6% of the total variation ($R^2 = 0.776$). This indicates a strong and non-random influence of the different strains on the multivariate composition of the rhizosphere microbiome. The table details the degrees of freedom (Df), sums of squares (SumsOfSqs), the pseudo-F statistic (F) value, the proportion of explained variance (R^2), and p-value derived from 9999 permutations (Pr(>F)), where a high F and low p-value signify significant differences attributed to the 'Strain' factor.

PERMANOVA	**Df**	**SumsOfSqs**	***F***	***R^2***	**Pr(>F)**
**Strain**	3	0.223161	23.061843	0.775748	0.001
**Residuals**	20	0.064511	NA	0.224252	NA
**Total**	23	0.287671	NA	1.000000	NA

Table S6. Pairwise PERMANOVA Analysis of Microbiome Differences Between Wildtype and T6SS Mutant Strains. Following a significant global PERMANOVA test, pairwise comparisons were performed to pinpoint specific strain pairs with distinct microbiomes. The wildtype strain exhibited significant differences from all mutant strains, as indicated by high F and *p-values* < 0.005. In contrast, comparisons between the mutant strains were not statistically significant after multiple comparison correction, suggesting greater similarity among the microbiomes of these groups.

Pairwise PERMANOVA	**Sample size**	**Permutations**	**F**	***p-value***
KT2440 vs *tssA1*	12	9999	3.247927	0.0030
KT2440 vs *tssM2M3*	12	9999	4.148943	0.0023
KT2440 vs *tssA1M2M3*	12	9999	2.199285	0.0021
tssA1 vs tssM2M3	12	9999	1.149619	0.1046
tssA1 vs tssA1M2M3	12	9999	1.373882	0.0729
tssM2M3 vs tssA1M2M3	12	9999	1.050389	0.1604

SUPPLEMENTARY REFERENCES

1. Hanahan D. Techniques for transformation of E. coli. In: Glover DM (ed.), DNA Cloning: A Practical Approach, IRL Press, McLean. Virginia, 1985, 109.

2. Herrero M, Lorenzo V de, Timmis KN. Transposon vectors containing non-antibiotic resistance selection markers for cloning and stable chromosomal insertion of foreign genes in gram-negative bacteria. *J Bacteriol* 1990;**172**:6557–6567. https://doi.org/10.1128/jb.172.11.6557-6567.1990 PMID - 2172216

3. Boyer HW, Roulland-Dussoix D. A complementation analysis of the restriction and modification of DNA in Escherichia coli. *J Mol Biol* 1969;**41**:459–72. https://doi.org/10.1016/0022-2836(69)90288-5 PMID - 4896022

4. Espinosa-Urgel M, Salido A, Ramos J-L. Genetic Analysis of Functions Involved in Adhesion of *Pseudomonas putida* to Seeds. *J Bacteriol* 2000;**182**:2363–2369. https://doi.org/10.1128/jb.182.9.2363-2369.2000 PMID - 10762233

5. Bernal P et al. The *Pseudomonas putida* T6SS is a plant warden against phytopathogens. *ISME Journal* 2017;**11**:972–987. https://doi.org/10.1038/ismej.2016.169

6. Kessler B, Lorenzo V de, Timmis KN. A general system to integrate *lacZ* fusions into the chromosomes of gram-negative eubacteria: regulation of the *Pm* promoter of the TOL plasmid studied with all controlling elements in monocopy. *Mol Gen Genet* 1992;**233**:293–301.

7. Kaniga K, Delor I, Cornelis GR. A wide-host-range suicide vector for improving reverse genetics in Gram-negative bacteria: inactivation of the blaA gene of Yersinia enterocolitica. *Gene* 1991;**109**:137–141. https://doi.org/10.1016/0378-1119(91)90599-7 PMID - 1756974

8. Vergunst AC et al. VirB/D4-Dependent Protein Translocation from Agrobacterium into Plant Cells. *Science (1979)* 2000;**290**:979–982. https://doi.org/10.1126/science.290.5493.979
